# A hypothesis on treatment strategy of severe multicentric Castleman disease with continuous renal replacement therapy

**DOI:** 10.1111/jcmm.70026

**Published:** 2024-09-09

**Authors:** Cătălin Constantinescu, David Kegyes, Bogdan Tigu, Vlad Moisoiu, Olga Grăjdieru, Andrea Szekely, Evangelos Terpos, Ciprian Tomuleasa

**Affiliations:** ^1^ Department of Hematology Iuliu Hatieganu University of Medicine and Pharmacy Cluj‐Napoca Romania; ^2^ Department of Anesthesia and Intensive Care Iuliu Hatieganu University of Medicine and Pharmacy Cluj‐Napoca Romania; ^3^ Intensive Care Unit Emergency Hospital Cluj‐Napoca Romania; ^4^ MedFUTURE Research Center for Advanced Medicine Iuliu Hatieganu University of Medicine and Pharmacy Cluj‐Napoca Romania; ^5^ Department of Anaesthesiology and Intensive Therapy Semmelweis University Budapest Hungary; ^6^ Department of Clinical Therapeutics, School of Medicine National and Kapodistrian University of Athens Athens Greece

**Keywords:** Castleman disease, continuous renal replacement therapy, intensive care unit

## Abstract

Castleman disease (CD) is a rare lymphoproliferative disorder, with non‐specific clinical manifestations, often delayed diagnosis and treatment, which pose a significant challenge in the present times. Patients diagnosed with this disease have poor prognosis due to the limited treatment options. Multicentric CD occurs at multiple lymph node stations and is associated with a proinflammatory response that leads to the development of the so‐called ‘B symptoms’. IL‐6 seems to be a key cytokine involved in various manifestations such as lymphadenopathies, hepatosplenomegaly, and polyclonal hypergammaglobulinemia. Its levels correlate with the activity of the disease. Other consequences of MCD include increased fibrinogen levels leading to deep vein thrombosis and thromboembolic disorders, high hepcidin levels causing anaemia, elevated VEGF levels promoting angiogenesis and vascular permeability, which, along with hypoalbuminemia, induce oedema, ascites, pleural and pericardial effusions, and in severe cases, generalized anasarca. In extreme cases multiple organ failure can occur, often resulting in death. We propose the use of continuous renal replacement therapy (CRRT) in managing severe multicentric CD. Our arguments are based on the principles that CRRT is able to remove IL‐6 from circulation thus attenuating the cytokine storm, can influence hepcidin levels, and reduction in oedema, and is often used in multiple organ failure to regain homeostasis control. Therefore, it could be used as a therapy or bridge therapy in severe cases. To sustain our hypothesis with evidence, we have gathered several studies from the literature confirming the successful removal of cytokines, especially IL‐6 from circulation, which can be used as a starting point.

## 1 BACKGROUND

Castleman disease (CD) is a diverse spectrum of lymphoproliferative disorders initially documented in the 1950s by Benjamin Castleman as a localized mediastinal tumoral mass mimicking a thymoma.^1^ Subsequently, clinicians discovered additional histopathological subtypes of the unicentric CD (UCD) over the following decades.^2^ In 1985, the systemic, multicentric form (MCD) has been also described.^3^ Diagnosis and treatment of patients with CD; however, still pose significant challenges in the present times.

## 2 EPIDEMIOLOGY

CD is classified as a rare condition. The incidence of CD is estimated to be approximately 21–25 cases per million person‐years worldwide.^4^ A recent study based on the introduction of a CD‐specific code in the International Classification of Diseases (ICD‐10) determined an incidence of 5.8 cases per million person‐years in the US.^5^ The incidence and prevalence of CD; however, can vary across different clinical studies, and there are also geographical and racial variations.^4^ CD can affect individuals of all age groups, including young children^6^ but typically, UCD patients are diagnosed at a younger age (in their 40s), while those with MCD are in their 50s–60s.^7^ Males tend to be slightly more affected by MCD compared to females, while no gender preference is observed for UCD.^7^ Human herpesvirus 8 (HHV‐8) is linked to nearly all HIV‐associated MCD cases and around half of HIV‐negative MCD cases.^8^

## 3 PATHOGENESIS

The pathogenesis of the two primary clinical entities CD (unicentric and multicentric) varies significantly. The aetiology of UCD is not thoroughly understood, recent genetic studies indicate that the clonal expansion of the stroma, particularly follicular dendritic cells may be involved. This expansion is believed to be driven by a gain‐of‐function mutation in the platelet‐derived growth factor receptor β.^9^ The genetics of CD; however, is far more intricate, as several other factors such as point mutations, karyotype abnormalities, and gene rearrangements may also play a role in the pathogenesis of CD. A comprehensive review of the genetic landscape of CD, both for UCD and MCD has been published by Butzmann et al.^10^ Additional next‐generation sequencing data, which were not covered in the previously mentioned review, have been also recently published.^11^ MCD, pathogenetically, is subclassified in human herpes‐virus 8 (HHV8)‐associated, POEMS (polyneuropathy, organomegaly, endocrinopathy, monoclonal plasma cell disorder, skin changes) syndrome‐associated and idiopathic MCD (iMCD). HHV8 infects B‐cells and plasmablasts, and within the lymph nodes, undergoes lytic replication. This process leads to the release of cytokines and the upregulation of signalling pathways, resulting in the induction of B‐cell and plasma cell proliferation, angiogenesis, and inflammation. In cases of POEMS‐associated MCD, the presence of a monoclonal plasma cell population leads to the production of various cytokines such as vascular endothelial growth factor (VEGF), interleukin‐6 (IL‐6) and interleukin‐12 (IL‐12).^7^ The levels of these cytokines correlate with the activity of the disease. The pathogenesis of idiopathic MCD (iMCD) is not well understood, and several mechanisms have been hypothesized. These include the synthesis of self‐reactive antibodies, oncogenic mutations, and germline mutations in genes related to inflammation, or infections. Regardless of the underlying mechanism, the common result is the release of cytokines and chemokines, either through their overproduction, the proliferation of B‐cells, or the overexpression of cytokine receptors.^12^ Activation of specific signalling pathways such as mTOR (mammalian target of rapamycin) and JAK‐STAT3 (signal transducer and activator of transcription 3) also play a major role in the induction of an inflammatory response.^13^ These processes ultimately lead to systemic manifestations. Carbone et al. and Fajgenbaum et al. comprehensively reviewed the driver pathogenetic events of CD.^14^, ^15^, ^16^

## 4 HISTOLOGY

UCD was previously classified into three distinct subtypes: hyaline‐vascular, plasma cell, or mixed‐UCD. However, because it is challenging to objectively differentiate between the plasma cell and mixed subtypes, the latest World Health Organization (WHO) Classification has merged them into a single category known as mixed/plasmacytic UCD.^17^ Approximately 65%–75% of biopsies reveal hyaline‐vascular UCD, while the remaining 25%–35% are classified as mixed/plasmacytic UCD. It is crucial to distinguish UCD from B‐ and T‐cell non‐Hodgkin lymphomas (such as mantle cell, follicular, marginal zone lymphoma, T‐cell lymphoblastic lymphoma), infectious diseases (HHV8 or mycobacterium‐associated infections), autoimmune disorders (systemic lupus erythematosus, Still's disease), and plasma cell neoplasms (plasmacytoma, lymphoplasmacytic lymphoma). A recent paper provides an excellent review of the histological features and differential diagnosis of UCD subtypes.^18^ MCD is histologically characterized by two common patterns: hypervascular and plasmacytic. However, in most cases, a mixed form is identified. The most common histological features of iMCD are regressed (or hyperplastic) germinal centers, plasmacytosis, hypervascularity, or prominent follicular dendritic cells. Wu et al. reviewed in detail the histologic diagnostic criteria of MCD.^19^

## 5 CLINICAL PRESENTATION

CD is now recognized as an umbrella term encompassing several distinct clinical syndromes and disease entities. Thus, the clinical presentation may vary from mild to severe, life‐threatening manifestations. Broadly, it is categorized into two main types: UCD and MCD. UCD is characterized by a slow‐growing, non‐malignant, painless solitary lymphadenopathy that initially remains asymptomatic. If symptoms are present they are usually caused by compression of the surrounding structures and are proportional to the size of the mass. MCD occurs at multiple lymph node stations and is associated with a proinflammatory response that leads to the development of the so‐called ‘B symptoms’. These symptoms include fever, night sweats, malaise, and weight loss. Elevated levels of IL‐6 result in various manifestations such as lymphadenopathies, hepatosplenomegaly and polyclonal hypergammaglobulinemia.^20^ The growth and differentiation factors produced by elevated IL‐6 levels affect both plasma cells and lymphocytes. Other consequences of MCD include increased fibrinogen levels leading to deep vein thrombosis and thromboembolic disorders, high hepcidin levels causing anaemia, elevated VEGF levels promoting angiogenesis and vascular permeability, which, along with hypoalbuminemia, induce oedema, ascites, pleural and pericardial effusions, and in severe cases, generalized anasarca. In extreme cases of iMCD, renal failure, hepatic failure or multiple organ failure can occur, often resulting in death.^21^ A systematic review reported the most common symptoms of iMCD being multicentric lymphadenopathy (100%), hepatosplenomegaly (78%), oedema, pleural/pericardial effusions or anasarca (78%), weight loss (72%) and fever (52%).^22^ Regarding lab findings, erythrocyte sedimentation rate (ESR) has been observed as the most frequently modified parameter (elevated in 92% of cases), followed by elevated IL‐6 (90%), hypoalbuminemia (90%), anaemia (87%), elevated C‐reactive protein (CRP) (82%) and hypergammaglobulinemia (77%).^22^ Depending on its clinical presentation, iMCD can be further subclassified into two subtypes: iMCD‐TAFRO and iMCD‐not otherwise specified (iMCD‐NOS). TAFRO syndrome was first described in 2010, and its typical manifestations are thrombocytopenia, anasarca, fever, reticulin myelofibrosis (or renal insufficiency), and organomegaly (hepatosplenomegaly and lymphadenopathy). Typically, iMCD‐TAFRO demonstrates a more aggressive disease course (lower platelet count, lower total protein, albumin levels, higher hepatic enzymes, blood urea nitrogen, and creatinine levels) and a worse response rate to immunosuppressive therapy.^23^ HHV8‐associated MCD and iMCD‐NOS have comparable clinicopathological features. However, iMCD‐NOS tends to exhibit a higher frequency of arthritis, cutaneous manifestations, renal disease, and lupus‐like symptoms in comparison to HHV8‐associated MCD.^7^

## 6 DIAGNOSIS

The diagnostic criteria of CD are summarized in Table 1. If CD is suspected, a comprehensive initial workup typically includes a thorough anamnesis, physical examination, complete blood count, erythrocyte sedimentation rate (ESR), C‐reactive protein (CRP) test, direct antiglobulin test (DAT), liver function tests, renal function tests, serum protein electrophoresis with immunofixation, and urinalysis.^7^ Imaging, such as computer tomography (CT) of the neck, chest, abdomen, and pelvis, or positron emission tomography (PET‐CT) and lymph node biopsy are required for diagnosis and further subclassification. In the case of MCD assessment of HIV and HHV8 status of the affected lymph node is also crucial. Elevated levels of IL‐6, as well as other markers such as soluble IL‐2 receptor (sIL2R), VEGF, IgA, IgE, lactate dehydrogenase (LDH) or β‐2‐microglobulin, are not pathognomonic to CD and their only role is to support the diagnosis. Nevertheless, exclusion of other autoimmune, infectious, and neoplastic conditions is mandated.^24^

**TABLE 1:** Diagnostic criteria of Castleman disease.

Subtype	Diagnostic criteria	
UCD	Localized lymphadenopathy in one lymph node station	
MCD‐HHV8	iMCD criteria	
	HHV8‐positive immunohistochemistry	
MCD‐POEMS	Major (all must be met)	Polyneuropathy
		Monoclonal gammopathy
	Minor (at least 1 must be met)	Sclerotic bone lesions
		High VEGF
		CD‐like or classical CD histology
		Volume overload (oedema, effusions, ascites)
		Organomegaly (spleen, liver, lymph nodes)
		Endocrinopathy
		Skin changes
iMCD	Major (all must be met)	Lymphadenopathies (≥2)
		CD histology
	Minor (≥2 must be met)	Constitutional B‐symptoms
		Hepatosplenomegaly
		Fluid overload
		Lymphocytic interstitial pneumonia
		Eruptive cherry hemangiomatosis
		Elevated ESR or CRP
		Anaemia
		Thrombocytopenia or thrombocytosis
		Hypoalbuminemia
		Renal dysfunction (eGFR <60 mL/min/1.73 m^2^ or proteinuria >150 mg/24 h)
		Polyclonal hypergammaglobulinemia
iMCD‐TAFRO	Histopathological	CD histology
		HHV8‐negative
	Major (3 of 5 must be met)	Thrombocytopenia
		Anasarca
		Fever
		Reticulin fibrosis
		Organomegaly
	Minor (at least 1 must be met)	Hyper/normoplasia of megakaryocytes
		High alkaline phosphatase without elevated liver enzymes
iMCD‐NOS	In case iMCD‐TAFRO criteria not met	

## 7 TREATMENT OPTIONS

CD is a rare disease, with minimum availability of therapeutic options. The effective treatment for UCD typically involves radical surgical removal, with or without preoperative embolization (recommended for highly vascularized lesions). Recurrence rates following surgery are generally low. In cases where the masses are deemed unresectable, adjuvant radiotherapy and immunotherapy (rituximab, siltuximab) should be considered as treatment options.^25^ A comprehensive international consensus‐based guideline for the treatment of UCD was published in 2020.^26^ Treatment of MCD starts with anti‐IL‐6 therapy, such as siltuximab, an anti‐IL‐6 antibody. Tocilizumab, an anti‐IL6‐receptor antibody is also tested in clinical studies but is not yet FDA‐approved for the treatment of iMCD. In siltuximab resistant MCD a histologic re‐evaluation should be performed to rule out transformation or misdiagnosis of a lymphoma prior to giving second‐line therapy. In case there is no response achieved, further drugs or drug combinations may be added to the IL‐6‐directed therapy, such as corticosteroids, rituximab (an anti‐CD20 monoclonal antibody), immunomodulators (thalidomide, lenalidomide), mTOR inhibitors (sirolimus) or calcineurin inhibitors (cyclosporine, tacrolimus).^27^ In the case of MCD‐POEMS, which is a very rare condition, characterized by a monoclonal gammopathy, standard myeloma treatment followed by an autologous transplant is recommended. IMCD, as opposed to MCD‐POEMS, is characterized by polyclonal hypergammaglobulinemia, where salvage therapies involving chemotherapy combinations may be used. If clinically possible, a histologic re‐evaluation to rule out the transition to or misdiagnosis of lymphoma is recommended in siltuximab‐resistant MCD before beginning any second‐line therapy. In the case of HHV8‐MCD, additional therapies such as interferon and antiviral therapy (such as ganciclovir, foscarnet, and cidofovir) might be added to the treatment regimen. A multidisciplinary approach to managing these individuals is always essential, regardless of the number of therapy lines or disease severity. There is also a need for novel biomarkers to measure therapy response and identify non‐responders early. CXCL13 seems to be a promising predictive biomarker of response to therapy in iMCD.^28^ Unfortunately, relapses and non‐responders do occur, and physicians should keep in mind that CD is not always a curable disease. The literature also mentions recurrent CD with increased serum levels of IL‐5 for which the response to rituximab is partial.^29^ As a result, efforts to find novel drugs and therapies to treat CD should be made.

## 8 PROGNOSIS

While the prognosis of UCD is favourable, most patients get cured by surgery, in the case of MCD, the situation is different. Liu et al. reported a 2‐year survival in iMCD of 93% (95% CI, 85–100), and progression‐free survival was significantly improved with siltuximab.^22^, ^30^ Proportions of patients with severe iMCD that require intensive care are around 10%–20% and present with a high risk of mortality.^26^ Longer follow‐up resulted in a 5‐year OS of 74% of MCD patients, significantly worse compared to UCD (*p* < 0.001).^30^ Multivariate analysis identified age >60 years, hepatosplenomegaly, anaemia, and hypoalbuminemia as negative prognostic factors.^31^ Vascular (thromboembolic disorders and inflammatory reactions) and renal complications are also associated with a poorer prognosis. Therefore, early diagnosis of these manifestations is necessary for tailoring appropriate therapy options, leading to a better prognosis. Also, novel treatment methods still need to be explored, especially in MCD. In the following, we hypothesize that continuous renal replacement therapy would be one possible, safe, and efficient way of bridging therapy in severe stages of MCD.

## 9 CONTINUOUS RENAL REPLACEMENT THERAPY (CRRT)

**FIGURE 1:**
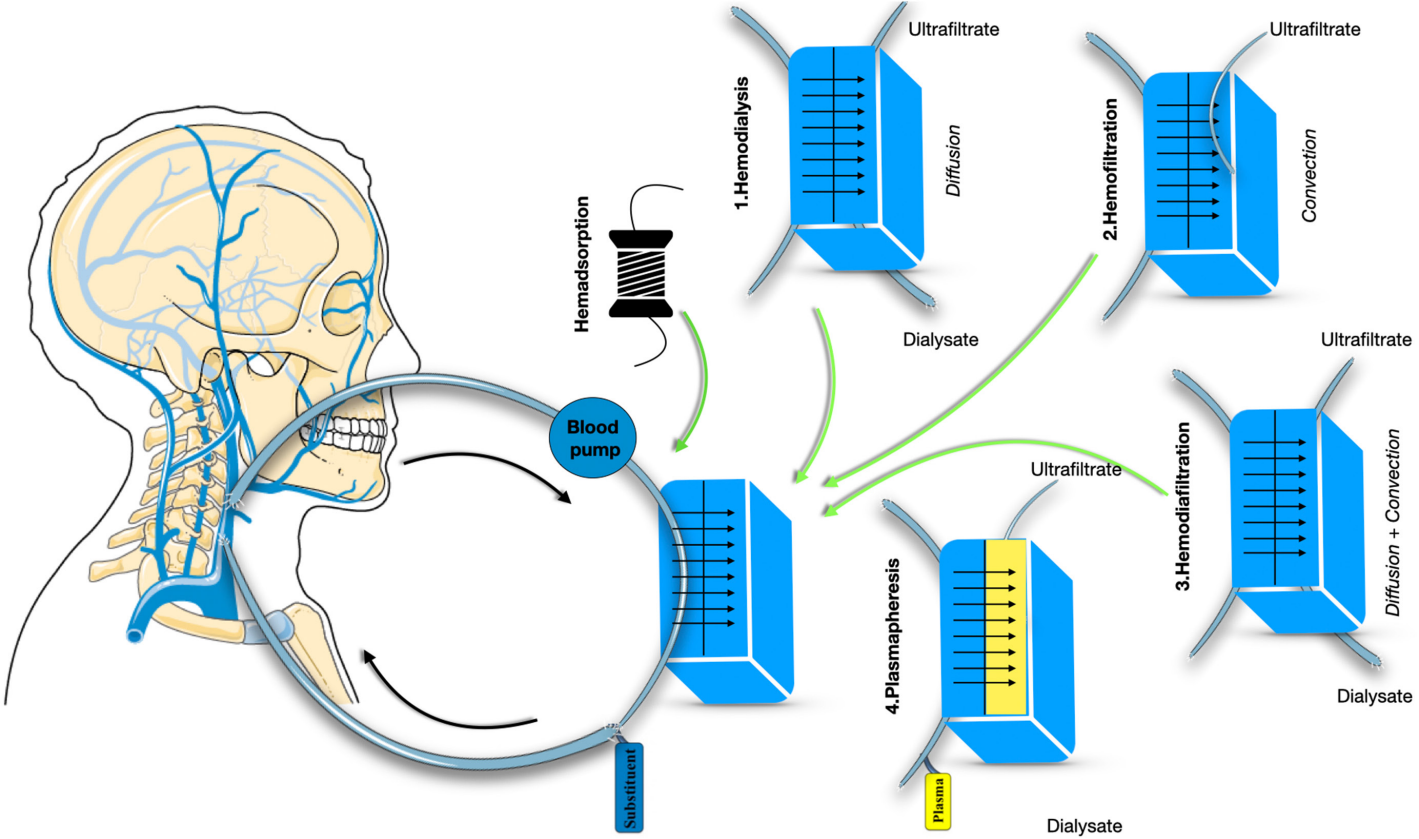
Different extracorporeal blood purification therapies. The blood passes through different filters with or without a hemadsorption cartridge and can undergo haemodialysis (1), hemofiltration (2), haemodiafiltration (3), or plasmapheresis (4) then the blood is recirculated into the bloodstream.

Continuous renal replacement therapy (CRRT) (Figure 1) is a type of extracorporeal blood purification technique, that is currently used in the intensive care unit (ICU) in patients suffering from multiple organ dysfunction syndrome (MODS), sepsis and septic shock, respiratory or cardiac failure, hepatic dysfunction, acute kidney injury, volume overload and other metabolic abnormalities.^32^, ^33^, ^34^ It offers the possibility of using dialysis and hemofiltration at lower blood pump rates, achieving a satisfactory removal of solute, and at the same time, it does not impact on the hemodynamics of the patients making the management of the critical patient easier.^35^ Moreover, CRRT can be used for the adsorption or removal of cytokines and other inflammatory mediators from circulation, by using different adsorption membranes, cartridges, or columns. They have been successfully employed in patients suffering from septic shock, in whom they decreased the plasma levels of proinflammatory cytokines and endotoxins. (Table 2).

**TABLE 2:** Studies assessing the removal of cytokines with the help of CRRT.

Study	No of patients	Membrane	Type of RRT	Cytokines
HCO membranes				
1.Park et al. (2016)^40^	212	Polyacrylonitrile AN 69 membrane	High‐dose CVVHDF	Reduced IL‐6, IL‐8, IL‐1B, and IL‐10 levels
2.Morgera S et al. (2003)^41^	16	PSH1 and Polyflux 11 S	iHP‐HF	Reduced IL‐6 levels, but poor for TNF‐α
3.Morgera S et al. (2004)^42^	24	PS2H	CVVH/CVVHD	Reduced plasma IL‐1ra and IL‐6 levels
4.Morgera S et al. (2006)^43^	30	PS2H and Polyflux 11S	CVVH	Reduced plasma IL‐1ra and IL‐6 levels
5.Atan R et al. (2016)^44^	26	PS2H and control	CVVH	Clearances were high for IL‐6 and IL‐8
MCO membranes				
6.Eichhorn T et al. (2017)^45^	30	Ultraflux EMiC2 (45 kDa) and Ultraflux AV1000S (30 kDa)	CVVHD	Removal of IL‐6 and IL‐8
7.Balgobin S et al. (2018)^46^	10	Ultraflux EMiC2 (45 kDa) and Ultraflux AV1000S (30 kDa)	CVVHDF	Mean levels of cytokines were not significantly different before and after RRT sessions
8.Lumlertgul N et al. (2021)^47^	12	EMiC2	CVVHD	All cytokines except EGF concentrations declined over 48 h
Hemadsobtion				
9.Malard et al (2018)^52^	Vitro	oXiris®, CytoSorb®, and Toraymyxin®		oXiris®–endotoxins and cytokines CytoSorb®–cytokines Toraymyxin®–endotoxines
10.Broman et al (2019)^53^	16	oXiris®	CVVHDF	Removal of endotoxins, TNF‐α, IL‐6, IL‐8 and INF‐y
11.Turani et al. (2018)^54^	60	oXiris®	CVVHDF	Decrease in IL‐6, IL‐10, endotoxin and procalcitonin
12.He et al. (2021)^12^	60	HA380	CPB	Reduced levels of IL‐1, IL‐6, IL‐8, IL‐10, and TNF‐α
Plasma exchange				
13. Nakae et al. (2002)^57^	26	Panflow APF‐06S and Plasmaflo OP‐08 W	TPE + CVVHDF	Reduced levels of TNF‐α and IL‐8
14. Knaup et al. (2018)^58^	20		TPE	Removal of IL‐6, IL‐1b, and angiopoetin‐2
15.Iwai et al. (1998)^59^	16		TPE	Removal of IL‐6 and TNF‐α

The cut‐off value of a membrane describes the smallest molecular weight of a solute that it withholds. High‐cut‐off membranes (HCO) have a value of around 65,000 Da (close to the value of albumin) and low‐flux membranes have cut‐off values between 10,000 and 30,000 Da.^36^ The elimination capacity for inflammatory mediators depends on the membrane used, but commonly used filters in CRRT have medium cut‐off membranes and achieve the elimination of solutes that have molecular weights between 30,000 and 50,000 Da.^37^, ^38^ Cytokines involved in the inflammatory and anti‐inflammatory pathways are small, having molecular weights between 8000 and 60,000 Da (Table 3). They are released from macrophages, B lymphocytes, T lymphocytes, mast cells, endothelial cells, fibroblasts, and stromal cells. The terminology overlaps but they are classified into interleukins (produced by leukocytes), monokines (produced by monocytes), lymphokines (produced by lymphocytes), and chemokines (produced by chemotactically active cells).^39^

**TABLE 3:** Clearance and adsorption of cytokines and endotoxines by the renal replacement therapies available.

	IL‐1 (63)	IL‐1*β*	IL‐2 (64)	IL‐3 (65)	IL‐4 (66)	IL‐6	IL‐8 (67)	IL‐10	IL‐12 (68)	IL‐13 (69)	IL‐17	INF‐y (70)	FGFs (71)	MCP‐1 (72)	TNF‐*α*	Endotoxin (lipopolysaccharide)	Procalcitonin (PCT)	VEGF
Molecular weight (kDa)	17	28	15.5	17	15	21–26	8	17	70	13	35	25	17–34	13	51	50–100	13	~20 monomeric form, ~40 dimeric form
	pro	pro	pro	pro	pro	pro	pro	anti	pro	anti	pro	pro	anti	pro	pro	pro	pro	pro
High cut‐off membranes × greater plasma protein losses																		
Theralite																		
Septex																		
Medium cut‐off membranes																		
Oxiris (35–40 kDa)																		
EMIC Ultraflux (~40–45 kDa)																		
AV600S (~30 kDa)																		
Hemoadsorbtion																		
HA380 (Jafron)																		
Cytosorb																		
Polymyxin B (Toraymyxin)																		
Coupled plasma filtration and adsorption (CPFA)																		

Abbreviations: FGF, fibroblast growth factor; IL, interleukin; INF, interferon; MCP, macrophage chemoattractive protein; TNF, tumour necrosis factor. The colour represents the cytokine that is removed by the respective filter.

### 9.1 The hypothesis

Based on the fact that CD involves the release of several cytokines, IL‐6 being one of the key ones, we have hypothesized that the use of CRRT in CD can be successfully used to remove circulating cytokines and improve the clinical outcome of these patients. Furthermore, CRRT can also be used as a comprehensive approach to the management of multiorgan dysfunction and to optimize the fluid balance which can change due to hypoalbuminemia. To document the removal of cytokines by the use of CRRT we have gathered several studies from the literature. (Table 2).

## 10 EVALUATION OF THE IDEA

### 10.1 Studies that have used high‐cut‐off membranes (HCO) membranes

A randomized controlled prospective trial conducted by Park et al. compared conventional (40 mL/kg/h) and high (80 mL/kg/h) doses of CVVHDF on 212 patients. High‐dose CVVHDF significantly reduced IL‐6, IL‐8, IL‐1B, and IL‐10 levels, but there were no differences observed in mortality between the groups.^40^ Morgera S et al performed two studies focusing on types of CRRT and the effect on cytokine clearance. In the first prospective, single‐center pilot trial, they performed intermittent high permeability hemofiltration (iHP‐HF) on 16 patients with multiple organ failure due to septic shock, with a polyamide hemofilter (PSH1, effective surface area 0.6 m^2^, 60 kDa cut‐off, steam‐sterilized, Gambro Corporate Research, Hechingen, Germany) alternated with conventional hemofiltration with a high‐flux polyamide membrane (Polyflux 11 S, effective surface area 1.1 m^2^, steam‐sterilized, cut‐off point approximately 30 kDa, Gambro Dialysatoren, Hechingen, Germany). The results concluded that there was a significant reduction in the total amount of circulating IL‐6, but the filtration capacity for TNF‐α was poor.^41^ In the second study, they compared CVVH with CVVHD by using an HCO membrane (P2SH; effective surface area 1.1 m2, steam sterilized; Gambro Corporate Research, Hechingen, Germany) on 24 patients with sepsis‐induced AKI, and concluded that increasing ultrafiltration volume or dialysate flow led to an increase in IL‐1ra and IL‐6 clearance rates (*p* < 0.00001) with a significant plasma level decline in those with high baseline levels. The clearance for TNF‐α was again, poor.^42^ This is also confirmed by another study performed by the same group.^43^ Atan et al. used a PSH2 hemofilter and found that the sieving coefficient values and clearances were high for cytokines like IL‐6 and IL‐8 using CVVH‐HCO but not CVVH‐standard.^44^

The mentioned studies suggest that HCO hemofiltration is able to successfully eliminate IL‐6 from plasma.

### 10.2 Studies that have used medium cut‐off membranes (MCO) (Ultraflux EMiC2 filter and oXiris®)

A study conducted by Eichhorn et al. confirmed the removal of IL‐6 and IL‐8 from plasma by using the Ultraflux EMiC2 filter,^45^ but contrary to this, a study performed by Balgobin S et al. using the same type of filter, showed that plasma levels of the proinflammatory cytokines TNF‐ α, IL‐1α, IL‐1β, IL‐2, IL‐6, and IL‐8, and the anti‐inflammatory cytokines IL‐4 or IL‐10, were not lowered during 24 h of CRRT.^46^ Moreover, a recent study by Lumlertgul et al. used CVVHD with the EMiC2 filter and detected a reduction in plasma levels of multiple cytokines during the first 48 h, but emphasized the fact that the in vivo clearance of cytokines may depend on other factors such as duration of blood contact with the filter, binding to protein or other plasma components, dialysate rate or ultrafiltration rate, molecular weight, serum concentration, or sampling time after filter installation.^47^ Going further, oXiris® hemofilter (Baxter, Deerfield, IL, USA) is a medium cut‐off polyacrylonitrile methalylsulfonate (AN69)‐based membrane technology. It has a surface coated with positively charged polyethyleneimine (PEI) and has permanent heparin on the inner membrane surface (4500 IU of heparin per m^2^). In addition to the renal supportive function attributable to the AN69 hemofilter membrane, the positively charged PEI coating enables the adsorption of negatively charged endotoxins in addition to cytokine adsorption and clearance.^48^, ^49^

CytoSorb® (Cytosorbents, NJ, US) is an extracorporeal cytokine hemoadsorption column, made of hydrophobic resin with small polymer beads (400–600 μm in diameter), which can remove cytokines in the range of (10–55 kDa) using its highly porous pyrrolidone‐coated polystyrene‐divinylbenzene polymer beads. It has a high removal rate of cytokines including IL‐6, IL‐10, TNF‐α, and INF‐y, based on the large surface area of the cartridge.^50^, ^51^

An experimental in vitro study conducted by Malard et al. compared three single‐use blood purification devices (oXiris®, CytoSorb®, and Toraymyxin®) for assessing the removal of cytokines and endotoxins. The endotoxin removal rate was similar for oXiris® and Toraymyxin®. The removal rate of pro−/anti‐inflammatory cytokines and other inflammatory mediators were similar between oXiris® and CytoSorb® and were higher with oXiris® and CytoSorb® vs. Toraymyxin.^52^ Moreover, Broman et al. confirmed the successful removal of endotoxins, TNF‐α, IL‐6, IL‐8, and INF‐y by using the oXiris® filter in 16 patients with septic shock.^53^

A retrospective study by Turani reported reductions in plasma levels of IL‐6, IL‐10, endotoxin, and procalcitonin with the use of an oXiris® filter in a case series of 60 patients.^54^

The hemocompatible cartridges HA130, HA230, HA330/380, (Jafron, Zhuhai City, China) are used for removing endogenous and exogenous materials such as middle uremic toxins, protein‐bound uremic toxins, hydrophobic or protein‐bound exogenous substances, cytokines, complements, free haemoglobin, and residual drugs by means of adsorption. They contain neutro‐macroporous resin adsorbing beads made of styrene‐divinylbenzene copolymer with an average diameter of the resin beads around 0.8 mm. The resin pore size ranges are 500 Da–40 kDa in HA130, 200 Da–10 kDa in HA230, and 500 Da–60 kDa in HA330.^55^ The HA380 cartridge has a range of 10–60 kDa, and there is confirmation that it is able to reduce the levels of inflammatory molecules, such as IL‐1, IL‐6, IL‐8, IL‐10, and TNF‐α, and correct the imbalance of inflammatory factors.^56^

### 10.3 Therapeutic plasma exchange (TPE)

Plasma exchange can remove molecules with high molecular weight, together with protein‐binding agents, endotoxins, and immune complexes. Plasma exchange can be used together with CVVHDF. A study conducted by Nakae et al. combined PE with CVVHDF and reported reduced levels of TNF‐α and IL‐8, but no difference in IL‐6 levels.^57^ Another prospective study enrolled 20 patients with septic shock and evaluated the effect of TPE on cytokine levels. It reported successful removal of IL‐6, IL‐1b, and angiopoietin‐2.^58^ TPE has also been used for the removal of TNF‐alfa and IL‐6 in patients with severe liver disease.^59^

### 10.4 Coupled plasma filtration and adsorption (CPFA)

The CPFA circuit consists of a plasma filter and a CRRT dialysis hemofilter connected in series. The plasma filter diverts the plasma of the patient through an adsorbent cartridge with a high affinity for inflammatory mediators (such as IL‐1, TNF‐α, IL‐6, IL‐8, C3a desArg, and IL‐10) and endotoxins. The filtered plasma is returned to the patient through a dialysis hemofilter for further solute and fluid removal.^60^, ^61^

### 10.5 CRRT effect on hepcidin and VEGF levels

Hepcidin has a crucial role in serum iron metabolism and appears to be correlated with IL‐6 levels which can induce the upregulation of hepcidin through the STAT‐3 signalling pathway.^62^ What is worth mentioning is that there is a report in which hepcidin levels have been reduced by the use of CRRT.^63^ Unfortunately, VEGF is a relatively large protein that is not typically removed by the filtration membranes used in CRRT. Any changes in VEGF levels observed during CRRT would likely be secondary to the treatment's effects on the patient's overall physiology and medical condition.

## 11 CONCLUSIONS

The management of patients with severe iMCD who fail to respond to the cytotoxic chemotherapy regimen or the anti‐IL6 therapy is not systematically specified. Thus, based on the pathophysiology of the disease and resemblance to other disease features, we suggest using CRRT as a bridge therapy in severe cases, to regain homeostasis control and to improve patients' outcomes. Well‐designed controlled clinical trials are required to clarify the role of CRRT in the possible treatment of MCD.

## AUTHOR CONTRIBUTIONS

**Cătălin Constantinescu:** Conceptualization (equal); funding acquisition (equal); writing – original draft (equal); writing – review and editing (equal). **David Kegyes:** Investigation (equal). **Bogdan Tigu:** Data curation (equal). **Vlad Moisoiu:** Methodology (equal). **Olga Grăjdieru:** Data curation (equal). **Andrea Szekely:** Data curation (equal). **Evangelos Terpos:** Visualization (equal). **Ciprian Tomuleasa:** Conceptualization (equal); data curation (equal); supervision (equal); validation (equal).

## FUNDING INFORMATION

This manuscript was funded by different grants. David Kegyes is funded by a grant from the Ministry of Research, Innovation, and Digitalization of Romania (Henri Coandă). Ciprian Tomuleasa is funded by an international grant from the European Haematology Association (EHA‐SWG Immunotherapy Project 2024—CAR NK cells for tumour‐associated macrophage immunomodulation—a new era of immunotherapy). David Kegyes, Bogdan Tigu, Vlad Moisoiu and Ciprian Tomuleasa are also funded by a bilateral collaboration grant between Romania and Moldova (PN‐IV‐P8‐8.3‐ROMD‐2023‐0036), as well as by a national grant of the Romanian Research Ministry (PNRR/2023/C9/MCID/I8) entitled ‘Creating a Research Group of Excellence to develop cell and immune therapy technology to target the tumor microenvironment’ (project code: CF 106/31.07.2024, contract number: 760278 /26.03.2024).

## CONFLICT OF INTEREST STATEMENT

The authors have no relevant affiliations or financial involvement with any organization or entity with a financial interest in or financial conflict with the subject matter or materials discussed in the manuscript. Ciprian Tomuleasa reports consultancy fees from EUSA Pharma Europe and Recordati Rare Disease Inc.

## Data Availability

All data are available, either analysed as figures and tables presented in the current manuscript or as raw data upon request by any external collaborator or reviewer. Figure 1 was partly generated using Servier Medical Art, provided by Servier, licensed under a Creative Commons Attribution 3.0 unported licence.
